# Ambulance attendance for substance and/or alcohol use in a pandemic: Interrupted time series analysis of incidents

**DOI:** 10.1111/dar.13453

**Published:** 2022-03-01

**Authors:** Rachael Mason, Amanda Roberts, Robert Spaight, Debbie Shaw, Gregory Adam Whitley, Todd E. Hogue, Aloysius Niroshan Siriwardena, Jim Rogers, Graham R. Law

**Affiliations:** ^1^ School of Health and Social Care College of Social Science, University of Lincoln Lincoln UK; ^2^ School of Psychology College of Social Science, University of Lincoln Lincoln UK; ^3^ East Midlands Ambulance Service NHS Trust Lincoln UK

**Keywords:** substance use, alcohol drinking, pandemic, emergency treatment, epidemiology

## Abstract

**Introduction:**

The ambulance attendance for substance and/or alcohol use in a pandemic (ASAP) study explores incidents during the COVID‐19 lockdown in the East Midlands region of the United Kingdom (23 March–4 July 2020).

**Method:**

Retrospective cross‐sectional count per day of ambulance attendances from the East Midlands Ambulance Service Trust. Ambulance attendances relating to alcohol or other drug use in the year prior, during lockdown and weeks following, were examined using interrupted time series analysis by patient demographics and geographical location.

**Results:**

A total of 36 104 records were identified (53.7% male, 84.5% ethnicity classified as White, mean age 38.4 years). A significant drop in the number of attendances per day at the start of lockdown (−25.24, confidence interval − 38.16, −12.32) was observed, followed by a gradual increase during the ongoing lockdown period (0.36, confidence interval 0.23, 0.46). Similar patterns were found across genders, age groups 16–64 and urban/rural locations.

**Discussion and Conclusion:**

The pattern of ambulance attendances for alcohol or other drug use changed during the COVID‐19 lockdown period. Lockdown significantly affected the use of ambulances for incidents involving alcohol or other drug use, impacting on health‐care services. Further research into hazardous use of alcohol or other drugs during the lockdown periods is needed to inform policy, planning and public health initiatives.

## Introduction

The SARS‐CoV‐2 epidemic resulted in an initial phase of ‘lockdown’ (where people were ordered to stay, and work, at home only leaving for essential purposes such as for food, medicine or daily outdoor exercise), causing people to face significant periods of time in their own home with limited social interactions starting 23 March 2020 in the United Kingdom (UK). The impact of quarantine is known to have many psychological implications [1] that influence health‐related behaviours such as alcohol or other drug use (AOD) [2, 3, 4].

Reports indicated the sale of alcohol across the globe increased during the first month of lockdown, with a continued increase during the second month in the UK [5]. The illicit drug market across Europe appeared to face problems with distribution at the consumer level, which resulted in a decrease of the purity of cocaine, heroin and amphetamine in some countries. This is in addition to a change in the market, for example, people substituting heroin for other substances and less demand for recreational ‘club’ drugs such as MDMA [6]. People who use illicit substances and/or alcohol may be at greater risk of experiencing severe symptoms of COVID‐19 due to poorer health and lower immunity, and pose a greater risk of transmitting the virus due to needing to travel to obtain substances [7, 8].

Concerns over alcohol use disorder, associated liver disease [9] and substance relapses [10] have been raised. Physical and psychological effects of alcohol withdrawal due to sudden lockdown have been reported [4, 11, 12]. Reviews of previous public health and economic crises suggest alcohol consumption may increase due to psychological distress, or decrease due to limited availability and financial constraints, differing by gender and socioeconomic status [13].

The Global Drug Survey COVID‐19 edition [14] reported the amount and frequency of alcohol use had remained the same for most respondents (males: 41%; females: 43%). More males reported drinking daily (7%) and having more than 10 drinks a day (6%) than females (4% and 3%), demonstrating the typical gender difference in alcohol use remained. The majority of respondents reported having not seen any harm reduction information during the pandemic (86%) suggesting there is a need to raise awareness of harms. The Crime Survey for England and Wales [15], which was conducted prior to the onset of COVID‐19, reported higher rates of substance use in younger people, in men (11.9%) compared to women (6.9%), in urban areas (9.6%) compared to rural (8%), and in lower income households (14.8% in households below £10 400).

Despite this, little is known regarding the effect of the COVID‐19 lockdown period on harms related to AOD, such as ambulance attendances. In this study, we examine the effect of the lockdown period of the UK on ambulance attendances for AOD in the East Midlands area, examining patterns in relation to patient demographics and geographical locations.

## Methods

### Design

A retrospective cross‐sectional analysis of the count of ambulance attendances per day over three time periods: year before lockdown (23 March 2019–22 March 2020), lockdown (30 March–3 July 2020) and weeks following the release of lockdown (4–31 July 2020). The outcome variable was the count of AOD‐involved attendances per day.

### Data source

Data on ambulance attendances at incidents across the East Midlands Ambulance Service Trust involving AOD from 23 March 2019 until 31 July 2020 were used. This included all attendances regardless of outcome (treat at scene, referral or transfer to hospital).

### Data extraction

Records were identified and anonymised by the East Midlands Ambulance Service Trust. Ambulance staff record details about the patient using clinical impression tick box categories, with additional clinical impression details in free text. Categories record most suspected incidents involving AOD. An additional search was conducted on the free‐text input using the following keywords determined by clinicians, researchers and the ethics committee. It is acknowledged this list was not exhaustive: narcotic, spice, mamba, alcohol, substance use, drug use, illicit drug, overdose, intoxication, intoxicated, drunk, high, solvents, solvent abuse, nitrous oxide, gas, aerosols, deodorants, petrol, under the influence.

### Variables

Time and date when the crew arrived on the scene was used to construct the main outcome variable, the count of attendances per day across the time series, with additional time variables related to the analysis specified below. Exposure data obtained from the attendance records per case consisted of patient age, gender, ethnicity and location postcode of ambulance attendance. Age was categorised into the following groups; under 16s, 16–24, 25–34, 35–44, 45–54, 55–64, 65–74, 75–84, 85+ years; gender into male, female and transgender; and ethnicity was coded into groups: White UK, Other White, South Asian, Other Asian, Black, Middle Eastern and Mixed/Other. Geographical location of the attendance was linked to Lower Super Output Area which are areas containing similar population sizes and as homogenous as possible based on tenure and accommodation type [16], categorised to Rural–Urban Classification based on population size (populations under 10 000 are classified as rural) [17], and Index of Multiple Deprivation that ranks all Lower Super Output Areas based on a combined measure of seven domains of deprivation; income, employment, education, skills and training, health deprivation and disability, crime, barriers to housing and services and living environment [18].

### Statistical analysis

We first identified a suitable time function to characterise the interruptions. The first national lockdown started on 23 March 2020 and was officially relaxed on 4 July 2020. Interruptions in the data were explored using B‐splines fitted to the count of the number of attendances per day. The splines were plotted against the time in days since lockdown, giving a polynomial function that describes the curve. Across the time length of 497 days, a knot was fixed every 7 days chosen given the well‐documented weekly patterns in AOD‐involved ambulance attendances [19, 20].

This was followed by an interrupted time series that was used [21] to model the number, or proportion, of attendances using ordinary least‐squared linear regression. A counterfactual scenario is imagined where the trend in the data would have continued without the interruptions. The counterfactual scenario provides a comparison for the evaluation of the impact of the intervention by examining any change occurring in the lockdown or post‐release period.

The outcome of the following exposure variables per model was count of number of attendances per day using linear modelling against:

- Time prior to (366 days) or elapsed since (103 days), 23 March 2020 [time].
- Monthly seasonality coded 1–12 [month].
- Day of the week coded 1–7.
- Variable indicating pre‐lockdown (coded 0) or after start of lockdown (coded 1), interpreted as the change in the level of the outcome at lockdown [interruption 1].
- The time in days since the start of lockdown indicates the slope change following lockdown [post interruption 1] coded 0 before lockdown and number of days since start of lockdown.
- Variable indicating time before the release of lockdown (coded 0) or after release of lockdown (coded 1), interpreted as the change in the level of the outcome on release of lockdown [interruption 2].
- The time in days since the release of lockdown indicates the slope change following release of lockdown [post interruption 2] coded 0 before the release of lockdown and number of days since release of lockdown.

Alcohol use varies according to time of year with use increasing in December around the festive period and in the summer months [22]. This seasonality effect was controlled by including a term for the month of year. Day of the week was also adjusted for in the model, given variations in alcohol consumption patterns across the week [23], and ambulance attendances [19, 20] being higher at the weekend.

Residual autocorrelation was tested by plotting the partial autocorrelation function by time lag. Based on these tests, the autocorrelation lag was adjusted using Newey‐West autocorrelation estimators for a 1 day lag [24].

Additional models were fitted separately stratified by levels of gender, geography, deprivation, age and ethnicity to explore any differences in the pattern of effects of the lockdown periods within the different levels of these variables.

All analysis was performed using Stata version 14 [25] and R [26].

### Patient and public involvement

A patient and public involvement group was developed for this project. They reviewed the study design and commented on research questions, methods and ethical considerations (see Supporting Information).

### Ethical approval

This study was given the National Health Service favourable ethical opinion (REC reference number 20/SC/0307) listed on the Integrated Research Application System as 286 198.

## Results

There were 780 782 ambulance attendances during the period 23 March 2019–31 July 2020. A total of 36 104 (4.6%) were incidents involving AOD: 6400 records during the lockdown period; 27 313 in the year prior; and 2391 in the weeks after release.

**Figure 1:**
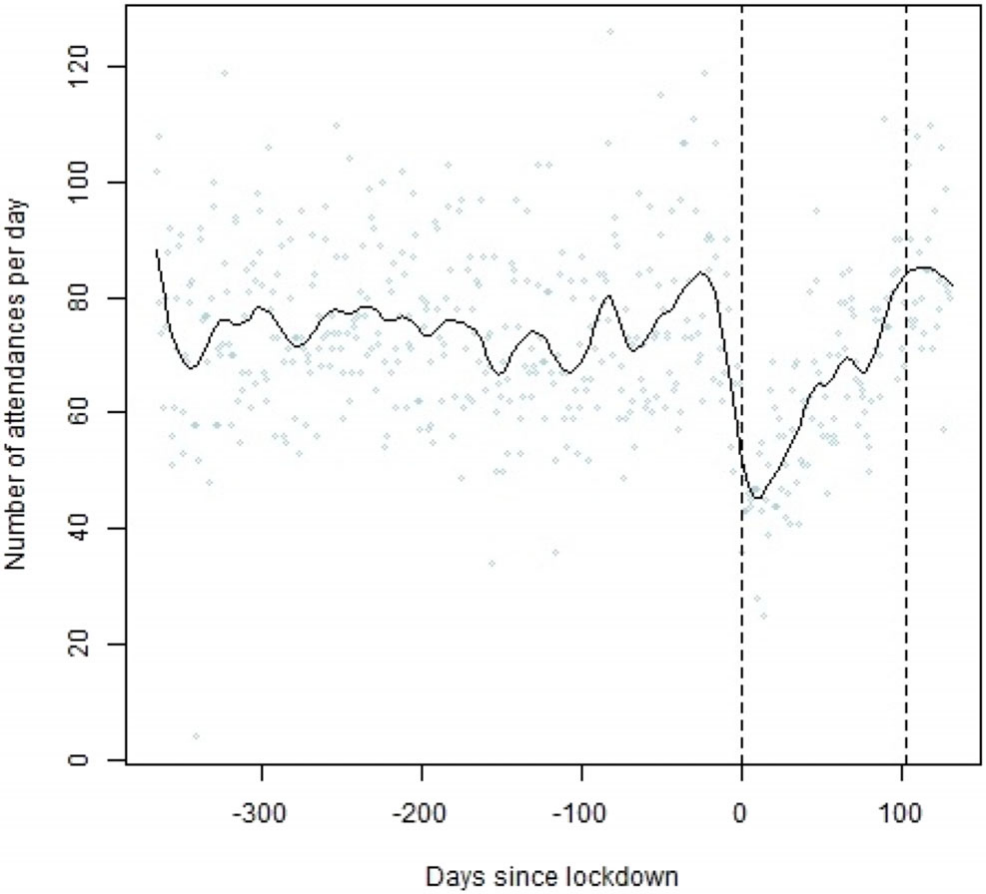
Smoothed B‐spline of alcohol/substance useattendances per day. Lockdown on 23 March 2020 (vertical line at day 0) and release on 4 July 2020 (vertical line at day 100).

Figure 1 shows the trend in average number of attendances per day relating to AOD over the full‐time period. There is a clear drop in the number of attendances shortly before the official time of lockdown with an increase in attendances after. The number levelled off and reduced slightly after the release of the lockdown.

Table 1 shows the number of ambulance attendances over the three time periods. Mean number of ambulance attendances per day prior to the introduction of lockdown was 1588.11, of which 74.63 (4.7%) were involving AOD. During the period of lockdown, the mean number of ambulance attendances per day was 1506.59, of which 62.14 (4.1%) were involving AOD. Immediately following lockdown, the mean number of ambulance attendances per day was 1594.04, of which 85.39 (5.4%) were involving AOD.

**Table 1:** Summary of sample

	Before lockdown	After lockdown until release	Release from lockdown	Total
Date		23 March 2020	4 July 2020	
Number of days	366	103	28	497
*All attendances*				
Number	581 250	155 179	44 353	780 782
Mean per day (SD)	1588.11 (130.27)	1506.59 (108.11)	1594.04 (68.34)	1570.99 (127.41)
*Alcohol/substance use attendances*				
Number	27 313	6400	2391	36 104
Mean per day (SD)	74.63 (14.99)	62.14 (15.26)	85.39 (13.15)	72.64 (16.05)

### Interrupted time series analysis

**Figure 2:**
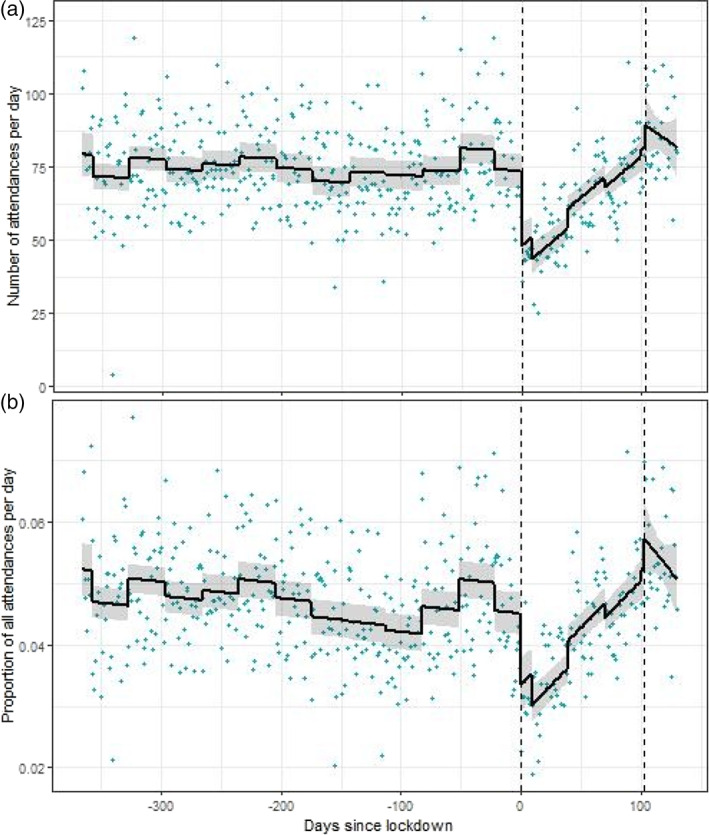
Interrupted time series (Linear modelling for interruption 1 (lockdown) on 23 March 2020 (vertical line at day 0) and second interruption (release) on 4 July 2020 (vertical line at day 100). Linear model: count = time + month + interruption 1 + post interruption 1 + interruption 2 + post interruption) plot of alcohol/drug use for (A) total count and (B) proportion compared to all attendances.

Figure 2 shows model‐predicted outputs from the interrupted time series model without day of the week for the attendances. Day of the week was excluded for the plot to give clarity for the pattern in relation to the interruptions. Figure 2A shows attendances per day involving AOD. Figure 2B shows the proportion of AOD‐involved attendances compared to all attendances.

Table 2 shows the coefficients for the interrupted time series models. Autocorrelation was tested with a plot of the autocorrelation factor by lag time in days. The plot (shown in Figure S1, Supporting Information) shows the autocorrelation for a lag of 1 is statistically significant. For all of the models, a lag of 1 was used.

**Table 2:** Coefficient (95% confidence interval) and *P* value using Newey–West estimator lag of 1 from interrupted time series model, for total attendances and attendances stratified by patient/attendance characteristics^a^

Model	First				Second			
	Step—initial lockdown date (95% CI)	*P* value	Post trend—initial lockdown period (95% CI)	*P* value	Step—date of lockdown release (95% CI)	Value	Post trend—following release (95% CI)	*P* value
Total	−25.24 (−38.16; −12.32)	**<0.001**	0.36 (0.23; 0.46)	**<0.001**	3.94 (−3.49; 11.38)	0.459	−0.48 (−0.80; −0.16)	0.074
*Gender*								
Female	−17.58 (−22.32; −12.85)	**<0.001**	0.20 (0.14; 0.26)	**<0.001**	3.81 (−2.54; 10.15)	0.2389	−0.43 (−0.75; −0.11)	**0.009**
Male	−7.63 (−12.71; −2.55)	**0.003**	0.16 (0.09; 0.22)	**<0.001**	0.76 (−6.05; 7.56)	0.827	−0.09 (−0.43; 0.26)	0.623
Trans	0.25 (−0.07; 0.56)	0.122	0.00 (−0.01; 0.01)	0.632	−0.08 (−0.50; 0.34)	0.716	0.01 (−0.01; 0.03)	0.402
*Geography*								
Urban	−21.75 (−28.95; −14.55)	**<0.001**	0.30 (0.21; 0.39)	**<0.001**	3.43 (−6.21; 13.08)	0.485	−0.39 (−0.87; 0.10)	0.119
Rural	−3.70 (−5.90; −1.50)	**0.001**	0.06 (0.04; 0.10)	**<0.001**	0.36 (−2.59; 3.31)	0.812	−0.10 (−0.25; 0.05)	0.197
*Deprivation*								
Most	−18.21 (−24.44; −11.97)	**<0.001**	0.24 (0.16; 0.32)	**<0.001**	0.44 (−7.90; 8.79)	0.917	−0.14 (−0.56; 0.28)	0.522
Least	−7.24 (−10.74; −3.74)	**<0.001**	0.12 (0.08; 0.17)	**<0.001**	3.44 (−1.34; 8.03)	0.162	−0.35 (−0.58; −0.11)	**0.004**
*Age, years*								
< 16	−0.29 (−1.33; 0.76)	0.592	0.02 (0.01; 0.04)	**<0.001**	0.51 (−0.89; 1.91)	0.473	−0.07 (−0.14; −0.00)	**0.044**
16–24	−10.14 (−13.84; −6.44)	**<0.001**	0.11 (0.08; 0.16)	**<0.001**	0.97 (−3.99; 5.92)	0.701	−0.12 (−0.37; 0.13)	0.349
25–34	−5.36 (−8.13; −2.58)	**<0.001**	0.04 (0.01; 0.08)	**0.013**	2.00 (−1.71; 5.72)	0.290	−0.11 (−0.30; 0.07)	0.238
35–44	−2.88 (−5.47; −0.28)	**0.030**	0.04 (0.00; 0.07)	**0.032**	−1.12 (−4.60; 2.36)	0.527	0.05 (−0.12; 0.23)	0.565
45–54	−3.21 (−5.81; −1.60)	**0.016**	0.08 (0.05; 0.11)	**<0.001**	−0.57 (−4.06; 2.93)	0.751	−0.04 (−0.21; 0.14)	0.679
55–64	−2.56 (−4.65; −0.47)	**0.016**	0.03 (0.01; 0.06)	**0.016**	2.30 (−0.49; 5.10)	0.106	−0.11 (−0.25; 0.03)	0.113
65–74	−0.08 (−1.36; 1.20)	0.903	0.02 (−0.00; 0.03)	0.063	−1.38 (−3.09; 0.34)	0.115	0.05 (−0.03; 0.14)	0.242
75–84	−0.44 (−1.27; 0.38)	0.291	0.01 (−0.00; 0.02)	0.090	0.56 (−0.54; 1.67)	0.318	−0.07 (−0.12; −0.01)	**0.015**
85 +	−0.08 (−0.51; 0.36)	0.734	0.00 (−0.00; 0.01)	0.194	0.28 (−0.31; 0.86)	0.348	−0.02 (−0.05; 0.12)	0.233

^a^ Linear multivariable models: count = time + month + day of week + interruption 1 + post interruption 1 + interruption 2 + post interruption.

*Note:* The *P* values that are in bold text are significant *P* values at the 95% confidence level. Those that are not bold are non‐significant *P* values.

At the first interruption, at the point of lockdown, there was a significant decrease in daily attendances of 25.24 (confidence interval − 38.16, −12.32). Following this, the number of attendances began to rise by 0.36 per day (confidence interval 0.23, 0.46). Apparent shifts in the trend following release from lockdown failed to reach statistical significance.

### Patient characteristics

#### Gender

Prior to lockdown, mean rates of ambulance attendance were higher for males (43.6 per day) than females (34.0 per day) that continued during the lockdown period and after release. Table 2 demonstrates that for both males and females, there was a significant step down in daily attendances at the point of lockdown followed by a significant increase until the point of national release. An increase in attendances for males and females continued at the point of release although these failed to reach statistical significance, followed by a significant decrease in the weeks following for females only. The data recorded more attendances for males than females over all three time periods (53.7% and 45.7%, respectively). Few attendances involved people identified as transgender (*n* = 93, 0.3%), precluding further analysis.

#### Age

Prior to lockdown, rates of ambulance attendance were highest for the age group 16–24 (17.6 per day). This was followed by the age groups 25–34 (13.4 per day), 35–44 (12.0 per day) and 45–54 (16.0 per day) years, and rates were lowest for 85 and overs (0.7 per day), followed by 75–84 (1.7 per day), under 16s (3.7per day) and 65–74 (4.0 per day) years. Table 2 demonstrates there was a significant step down in attendances at the point of lockdown in the age categories 16–24, 25–34, 35–44, 45–54 and 55–64 years. These age groups saw a significant increase in attendances during the lockdown period although there was less of a difference between the 16–24 age group compared with 25–34, 35–44 and 45–54 years during the lockdown period in contrast to pre‐lockdown. A similar pattern of attendances was observed over the majority of the age groups following the national release (a continued increase) and the few weeks following (a decrease) although fewer results reached statistical significance.

#### Ethnicity

The ethnicity of patients for the large majority of attendances (84.5%) was recorded as White, with the remainder distributed in small numbers across a range of different ethnicities, precluding detailed analyses of these ethnic groups.

### Attendance characteristics

#### Rural versus urban

The number of attendances over the three‐time periods was larger within urban areas (31 402) compared to rural areas (4596). Table 2 demonstrates that attendances in both types of areas declined significantly at the point of lockdown but increased significantly during the lockdown period. Apparent shifts in the trend following release from lockdown failed to reach statistical significance. The coefficient was larger for the urban areas due to the number of attendances.

#### Deprivation

The number of attendances over the three time periods was higher within the 50% most deprived areas (25 928) compared to the 50% least deprived (10 070). Table 2 demonstrates that attendances in both the most and least deprived areas declined significantly at the point of lockdown but increased significantly during the lockdown period. An increase in attendances for both areas continued at the point of release although these failed to reach statistical significance, followed by a significant decrease in the weeks following for the least deprived areas only.

## Discussion

This study found the number of ambulance attendances involving AOD in the East Midlands significantly decreased at the initial point of the UK's national lockdown, followed by a gradual but significant increase during the lockdown period, eventually returning to the rate of attendances pre‐lockdown. At the point of national release from lockdown, ambulance attendances in the East Midlands did not change significantly, but the period we examined post release was only short. Patterns of attendance were similar across genders, the age groups 16–64, geographical locations (urban versus rural) and areas of deprivation.

Although the average number of daily ambulance attendances to incidents involving AOD remained consistent during the lockdown period compared to the previous year, the interrupted time series analysis illustrates the nuance of the pattern that emerged within the data set. There was an abrupt drop in the number of ambulance attendances per day for any incidents following the implementation of the national lockdown in the East Midlands (13%—data not shown) similar to findings [27] that attendance at UK‐based emergency departments decreased in the first month of the pandemic (March 2020) across all patient demographics and conditions. The pattern in our data was more prominent for incidents involving AOD with data illustrating a larger drop in ambulance attendances (around 33%), although this could have been due to the different settings for each of the studies (emergency department attendances vs. ambulance attendances). During the period of lockdown, the daily number of ambulance attendances began to rise, marginally surpassing pre‐lockdown levels. Data for attendances involving AOD demonstrated a significant increase following the initial drop with rates returning to what was experienced prior to lockdown. During the lockdown, the ambulance service will have been required to respond to this rise in attendances for AOD.

A study in the United States exploring rates of ambulance calls relating to substance use during the pandemic [28] demonstrated similar results. Weiner *et al*. [28] found rates of calls decreased for substance use after a state‐wide national emergency was declared for COVID‐19, followed by a significant increase that was not seen for other conditions. This suggests our findings may be indicative of a wider global occurrence, and that COVID‐19 restrictions are having a disproportionate effect on those who use substances. Calls for ambulance attendance rather than actual attendances were used in Weiner *et al*.'s [28] study, which may impact on comparing the results as calls for ambulance attendance may have been higher than actual attendance. A further study in Kentucky, USA, explored rates of ambulance attendance for opioid overdoses following the state‐wide national emergency [29]. Their findings illustrated the increase in opioid overdoses following this declaration resulting in a 17% increase in the number of attendances with subsequent transport to hospital, a 71% increase in attendances where transportation to hospital was refused, and a 50% increase where the attendance was associated with a death at the scene. Our study did not differentiate between the reason for the attendances involving AOD and further analysis on the outcome of attendance, or the specific reason for attendance, may help to understand the behaviours further.

The data suggest that there is a distinct pattern for attendances involving AOD that differs from the overall ambulance attendance data. The abrupt drop in attendances may have been due to the closing of nightlife venues such as bars and clubs, availability of substances and/or the general public's fear of calling an ambulance during a national pandemic as suggested in previous studies [28, 30]. The increase however, may demonstrate the issue of hazardous AOD that may occur due to lockdown enforcement resulting in lack of social connection, isolation, financial and occupational stress, and overall psychological distress [4, 12] as well as the potential change in substance use or purity of these during the pandemic. However, this study did not examine how or why AOD use was occurring and further exploration is needed to examine any connection. The rate of increase during the lockdown at which the ambulance service was required to attend to incidents relating to AOD demonstrated the ongoing impact of AOD on this service during this time.

The pattern of ambulance attendances was similar for males and females, with a slightly larger coefficient for the initial decrease during the lockdown period for males from a higher starting point during the pre‐lockdown period. This finding is consistent with the numbers of males receiving treatment for alcohol use (61%) and substance use (73%) is higher than females (39% and 27%, respectively) [31], highlighting how higher levels of consumption among males [^15^] flow through to harms such as ambulance attendances and treatment for dependence.

The pattern of AOD use was similar across the age groups 16 to 64, with those aged 16–24 years having the highest levels of ambulance attendances and the sharpest increase during lockdown, followed by 25–34 and 35–44. This highlights how ambulance attendance for different age groups is consistent with those in treatment. The age group with the highest number of people in treatment for AOD in 2016/2017 was 35–39 years, with non‐opiate use tending to be younger (25–29 years) and alcohol use tending to be older (45–49 years) [12]. Our data suggest harmful use may therefore occur at a younger age, before entering treatment based on the figures shown for this and may place additional stress on the ambulance service due to no known support systems/treatment plans in place for the individual.

Problematic drinking occurs more frequently in the highest‐earning households [32] although substance‐related harms are greater in more deprived areas [33]. We found ambulance attendances for incidents involving AOD occurred most frequently in urban areas and in the most deprived locations. This highlights the importance of understanding the wider determinants of health and the impact geographical and economic factors may have on AOD [33], alongside the need to offer support for the most vulnerable during pandemics.

### Strengths and weaknesses

This study utilises data from the East Midlands, a large area of the UK, serving over 4.8 million people which may nonetheless not be generalisable to other counties.

Due to the retrospective nature of this study, cases may have been missed as clinicians may not have documented the clinical impression or used the terminology searched for in the free text. The free‐text search terms were not exhaustive and did not account for spelling errors which may have resulted in relevant records being missed.

The smoothing of the data using B‐splines used a 7‐day gap between knots. This was chosen to hopefully reflect the weekly patterns in the data although this may have prevented identification of any patterns outside of this. The ethnicity of patients was predominantly white (84.5%) and there were limitations with how this data was recorded. Further research would require more accurate recording of ethnicity with a larger data set to capture more ethnically diverse populations. There are limitations in the definitions of rural and urban, as well as deprivation, which may impact on our results. Government guidelines were used for determining these areas.

These results suggest a significant impact on AOD requiring ambulance attendance during lockdown in the East Midlands region of the UK. The initial decline suggests there may have been a change in behaviour or in ambulance service response. The increase in attendances during lockdown suggests it may have impacted on hazardous AOD. The reasons for this are still to be explored although may be linked to the psychological and economic distress caused by the pandemic, the availability of substances, and the fear of using health services. Those who live in urban and more deprived areas are likely to request ambulance attendances at a greater rate than those in rural and less deprived areas [15]. This may be useful for planning of ambulance care in relation to geographical need, and training for ambulance crew responding to AOD incidents. Those aged 16–24 years requested ambulance attendance more than other age groups and more awareness of this group in relation to AOD may be helpful for ambulance staff.

We aimed to explore any differences in ethnicity of the attendances as the East Midlands Ambulance Service Trust covers a large population, and COVID‐19 was reported to have a differing impact on different ethnic groups. The East Midlands has a high proportion of people reporting their ethnicity as white (85.4%) [34] and our data regarding incidents involving AOD appears to reflect this, with 84.5% of incidents recording the ethnicity of the patient as white. The recording of patient ethnicity was completed in free text form by the attending ambulance crew and self‐reported by the patient. This may have created inaccuracies in our groupings and several records did not record the ethnicity of the patient creating further obstacles with this variable and preventing further analysis.

### Unanswered questions and future research

Our study does not provide explanations for why ambulances were called in these cases, nor insight into the lived experience of people who use substances and alcohol which would provide further insight into AOD during the pandemic. The post‐lockdown release data gathered was over the period of a few weeks; to examine longer‐term impacts of the pandemic, data from a longer time period are required. Exploring the impact of subsequent lockdowns and releases would determine if the trend in these results is corroborated. Our data did not differentiate between alcohol and other substances and our results may be influenced by the trend in incidents involving alcohol. Further analysis which explores the number of attendances for different substances may illustrate any patterns based on this variable. Following the patient pathway through from the initial ambulance attendance will illustrate the system approach to AOD in a pandemic, allowing a deeper understanding of this behaviour and its impact on other services.

## Conflict of Interest

The authors have no conflicts of interest.

## Supporting information


**Appendix** S1. Patient and public involvement and engagement (PIPE).

**Appendix** S2. STROBE Statement—Checklist of items that should be included in reports of cross‐sectional studies.

**Figure S1.** Partial autocorrelation factor plot by lag in days.

## Data Availability

The authors agree to the sharing of the anonymous count data using ePrints.
