# Supplementary material for: Ambulance attendance for substance and/or alcohol use in a pandemic: Interrupted time series analysis of incidents
Source: Drug Alcohol Rev. 2022 Mar 1;41(4):932–40. doi: 10.1111/dar.13453 (PMC9111577; doi:10.1111/dar.13453)
Supplement: Supplementary file 1 — Appendix S1. Patient and public involvement and engagement (PIPE). [file DAR-41-932-s002.docx]

**Appendix S1. Patient and public involvement and engagement (PIPE)**

Study: *Ambulance attendance for substance and/or alcohol use in a pandemic (ASAP): Interrupted time series analysis of incidents.*

**PPIE members:**

1. Public representatives (n=2)
2. Drug and alcohol misuse charity representatives (n=1)
3. Registered paramedic (n=1) [PPIE Lead]
4. Paramedic students (n=5)

PPIE members were sent a “consultation document”. This summarised the proposed research project and methods. The following pertinent questions were asked:

1. Do you think the research is important?
2. Do the research questions make sense?
3. Is anything unclear?
4. Have we missed anything important?
5. What outcome is important for patients attended by ambulance who have taken alcohol / substances? (in other words, what is a good measure of the quality of care? For example transport to hospital? Referral to other services/GP? Extended time on scene? Completion of alcohol/substance risk assessments?)

PPIE members were encouraged to offer additional comments and feedback.

# Feedback received

## Public representative #1

Hi... Thank you for sharing your document, which I found interesting to read and consider. I have given some thought to your questions and offer the following observations and thoughts to the questions you pose...

1. Yes I think the research is important in terms of identifying and categorising appropriate treatment approaches and potentially keeping people out of hospital.

2/3. You refer to attendance at incidents involving alcohol/substance abuse... are the incidents simply an individual calling 999 and telling the call handler they've taken xyz... or are the incidents related to the consequences of alcohol/substance… eg violence/accident/collapse etc etc.

In terms of the factors predicting whether an ambulance is dispatched... might this also be influenced by who has made the call...the individual or an on-looker/friend/relative... and does the identity of the caller affect the answers given to the call handler?

4 Have you missed anything... is there something about why ambulance staff might alter clinical practice during a pandemic... is it additional risk... pressure on beds...??

5 A good outcome... if its just alcohol/substance abuse rather than consequence... injury/collapse etc... I think i would vote for referral to appropriate service.

## Public representative #2

I have had a look through this and it looks a good project. I have a few comments (see below) which you may wish to consider.

1) Comparison of response over 16 months in previous years. I wonder if it would be worth looking at say two years for the same period as control as one year might be abnormal?

2) Is it possible (if recorded) to include the socio-economic status of the subjects under study?

3) Is it worth considering if calls were made by the patient or a "friend" if this is known?

4) Does the recorded information show if there are repeat calls from the same person?

5) Do you have the information as to whether the patient showed signs/symptoms of COVID ?

6) Re crew gender - will not many of the crews be mixed gender and is this a relevant factor?

7) Need to note if any of the crews have had special training re mental health issues.

## Drug and alcohol misuse charity representative

The PPIE lead and the representative discussed the ASAP PPI consultation document. Several comments were made regarding the ASAP study, summarised below:

The research topic and questions were of high importance.

The use of anonymised data was acceptable but not ideal, as it limits traceability, and we will not be able to follow patients through their journey.

It would be interesting the test the association between reduced/altered service function, such as reduced/altered naloxone and methadone supply and any subsequent increase in rates of overdose across the East Midlands. The same would apply for alcohol services and patients suffering subsequent withdrawal symptoms.

For example, within *anonymised* the distribution of naloxone has changed, and service users now need to book an appointment (instead of a drop-in service) or attend a pharmacy. Some service users may not wish to do this. This may have caused an increase in rates of OD. This knowledge would be of public interest and useful for commissioners.

Unsure about how other services have been affected across East Midlands.

## Paramedic student representatives (cohort student representative n=1)

*Involving the student voice:*

From a Student rep perspective if this was my research project I would email all students in the cohort (Bcc) asking if anyone wanted to give their feedback on the project then on replying with their interest email them the document and a google survey or some form of anonymous survey platform to gain feedback. I also think that using a survey platform and an expression of interest would prevent the document being unnecessarily shared to all students and would allow for only those genuinely interested in giving their feedback to participate and access the survey.

*What do you think of the project idea - general comments and feedback?*

Overall, I believe this is a really good idea, it covers a key issue that paramedics deal with which is alcohol/substance misuse and through covering the entirety of EMAS and all calls following the given key words there’s a really clear valid participant group included rather than just picking one station or call centre for example.

*Would a project like this be useful for paramedics and why?*

I absolutely believe this would be a useful project for Paramedics as it gives quantitative insight into a possible increase/decrease of calls regarding substance and alcohol misuse but also quantitative knowledge about other paramedic’s care pathways in terms of admitting or avoiding hospital due to the COVID-19 outbreak.

*Would a project like this be useful to the public and why?*

I’m sceptical if this would be useful for the general public as its more applicable to pre-hospital regarding call numbers and care pathways however I do think that focusing on gender, age, ethnicity and location would be useful for the public or potentially other professionals to see any target groups/areas to focus on when signposting for alcohol or substance misuse.

*What changes would you make to the project and why?*

I wouldn’t make any big changes, but I would consider interviewing a control group of paramedics or call handlers about their covid-19 experiences relating to the type of calls being examined. Although it will be relatively personal and potentially bias, I think it would be insightful to speak to EMAS staff from various areas of the region to see if their experiences have differed. E.g. have paramedics that cover a notably ‘affluent’ area of the region had different experiences to those that cover potentially lower socio-economic areas.

*Are there any other questions you think we could ask?*

Although it broadens the research aim/questions wider I would be interested form a Student Paramedic Point of View to see how many of these calls with the given key words then went on to have a safeguarding referral made for them by the attending crew. I think this would highlight the increase of vulnerability for callers that suffer from alcohol or substance misuse.

*Are there any additional ethical concerns for us to consider?*

I agree with all the ethical points given especially the consideration of the patient data being collected without consent. I don’t have any immediate concerns the only additional consideration I may make is potential harm or distress results may cause to certain population groups if a trend is identified in calls e.g. a particular ethnic group or if a frequent alcohol/substance misuse caller from the EMAS area reads the project and feels shame/reluctance to call in the future, however I accept that these are very small possibilities and the ethical considerations given in the document cover the main principles of anonymity and the need for research.

## Paramedic student representatives [student responses n=4]

# PPIE Feedback Actions

The study team used routinely collected data, therefore many of the PPIE comments regarding the analysis of additional data were unable to be incorporated, however these comments will be considered for future research.

A pertinent comment, from the drug and alcohol misuse charity representative was around the alteration of services due to COVID-19 and any subsequent impact on patient outcomes. Unfortunately, as part of this study were unable to test this association as it was beyond the scope of the project. Again, this is something that would be interesting to assess during further research.

Public representative #1 raised a comment regarding the nature of 999 call; we included clinical data where the clinical impression was related to alcohol or drug misuse, therefore this categorisation was dependent on the attending clinician’s working diagnosis. Public representative #2 commented about the socioeconomic status of patients and asked whether this could be assessed. This feedback was translated into the study as we ensured that the association between deprivation and alcohol and drug misuse during the COVID-19 pandemic was assessed. The paramedic student feedback was overall positive and supportive of the study and corroborated other comments stating that the study was important and the findings of the study would be useful. The student comment about using data from more ambulance services was useful and again will be considered for future research.

Overall, the PPIE members concluded that the topic of research was important and the research question made sense. Feedback and comments were incorporated into the study where possible and considered for future research where not possible.
