# Supplementary material for: Ambulance attendance for substance and/or alcohol use in a pandemic: Interrupted time series analysis of incidents
Source: Drug Alcohol Rev. 2022 Mar 1;41(4):932–40. doi: 10.1111/dar.13453 (PMC9111577; doi:10.1111/dar.13453)
Supplement: Supplementary file 3 — Figure S1. Partial autocorrelation factor plot by lag in days. [file DAR-41-932-s003.docx]

**Supporting Information**

**Figure S1. Partial autocorrelation factor plot by lag in days**
